# TMEM106B aggregation in neurodegenerative diseases: linking genetics to function

**DOI:** 10.1186/s13024-023-00644-1

**Published:** 2023-08-10

**Authors:** Hai-Shan Jiao, Peng Yuan, Jin-Tai Yu

**Affiliations:** 1 Department of Neurology and National Center for Neurological Disorders, Huashan Hospital, State Key Laboratory of Medical Neurobiology and MOE Frontiers Center for Brain Science, Shanghai Medical College, Fudan University, Shanghai, 200040 China; 2 Department of Rehabilitation Medicine, State Key Laboratory of Medical Neurobiology, MOE Frontiers Center for Brain Science, Huashan Hospital, Institute for Translational Brain Research, Fudan University, Shanghai, China

**Keywords:** TMEM106B, Amyloid fibrils, Aggregation, Neurodegeneration, Lysosome, Therapeutics

## Abstract

**Background:**

Mutations of the gene *TMEM106B* are risk factors for diverse neurodegenerative diseases. Previous understanding of the underlying mechanism focused on the impairment of lysosome biogenesis caused by TMEM106B loss-of-function. However, mutations in *TMEM106B* increase its expression level, thus the molecular process linking these mutations to the apparent disruption in TMEM106B function remains mysterious.

**Main body:**

Recent new studies reported that TMEM106B proteins form intracellular amyloid filaments which universally exist in various neurodegenerative diseases, sometimes being the dominant form of protein aggregation. In light of these new findings, in this review we systematically examined previous efforts in understanding the function of TMEM106B in physiological and pathological conditions. We propose that TMEM106B aggregations could recruit normal TMEM106B proteins and interfere with their function.

**Conclusions:**

*TMEM106B* mutations could lead to lysosome dysfunction by promoting the aggregation of TMEM106B and reducing these aggregations may restore lysosomal function, providing a potential therapeutic target for various neurodegenerative diseases.

## Background

Mutations of *TMEM106B* have been identified as genetic risk factors for neurodegenerative diseases including Frontotemporal lobar degeneration (FTLD) [1] and limbic-predominant age-related TAR DNA binding protein 43 (TDP-43) encephalopathy [2]. TMEM106B has also been reported to modulate patients’ cognitive functions in other neurodegenerative diseases, such as Alzheimer’s Disease (AD) [3], Parkinson’s Disease (PD) [4], and amyotrophic lateral sclerosis (ALS) [5]. The classic studies of TMEM106B function indicated that this protein is an important regulator for lysosomal function [6]. Thus, the disease-related *TMEM106B* genetic polymorphisms could contribute to pathogenesis by disrupting lysosome functions. This view has recently been challenged by a series of reports. These studies showed that TMEM106B aggregation is a widespread pathology that exists in the post-mortem brain tissues of diverse neurodegenerative diseases, including FTLD, PD, AD, ALS and multiple system atrophy (MSA) [7–10]. These findings suggest that TMEM106B can form protein aggregation that may be contributing to the neurodegeneration. The collection of these studies points out that the underlying mechanism linking *TMEM106B* mutation and disease onset could be more than the loss-of-function for regulating lysosome biogenesis, but could be a gain of toxicity due to the formation of protein aggregates. In light of this new possibility, a re-evaluation of previous opinion of TMEM106B’s function and association with neurodegenerative diseases is needed.

## Main text

### *TMEM106B* is associated with clinical characteristics of neurodegenerative diseases

#### Mutations in *TMEM106B* are risk factors for diverse neurodegenerative diseases

Human *TMEM106B* gene is located on chromosome 7p21, with nine exons. The most well studied single-nucleotide polymorphism (SNP) rs1990622 is in a non-coding area that could play a regulatory role. The T allele at this position is considered the major isoform (T/C frequency is 0.58/0.42 in Caucasian population and 0.37/0.63 in Asian) [11]. The major T allele has been linked to higher risks for developing neurodegenerative diseases or exacerbated cognitive decline, whereas the minor C allele is associated with a protective phenotype. In addition, one coding variant of *TMEM106B*, Thr185Ser encoded by SNP rs3173615 (C/G 0.60/0.40 in Caucasian and 0.37/0.63 in Asian), has been reported to be protective against several neurodegenerative disorders [12, 13]. We summarized these human association studies below.

The most robust associations between *TMEM106B* polymorphism and the development of diseases have been reported in diseases in which TDP-43 is the major proteinopathy in the brain. For example, TDP-43 inclusion bodies are the primary aggregation found in a major subtype of frontotemporal lobar degeneration (FTLD-TDP) patients. Also, genome-wide association studies found that the major T allele of SNP rs1990622 was linked to an increased FTLD-TDP risk (odds ratio: 1.64), whereas the minor C allele was protective (odds ratio 0.61) [14–16]. The allele rs1990621 has been reported to be associated with neuroprotective effects among FTLD patients [3]. Among people carrying Progranulin gene (*GRN*) mutations, which are known to cause FTLD, *TMEM106B* SNP rs1990622 could further increase the risk for FTLD-TDP, potentially by modulating GRN levels [14, 15, 17]. On the other hand, studies found no relationship between rs1990622 and subtypes of FTLD without the TDP-43 pathology [18], suggesting that the interaction with TDP-43 could be important for the pathogenesis effect of *TMEM106B* SNPs.

Consistent with this view, Limbic-predominant age-related TDP-43 encephalopathy (LATE) is another disease with prominent TDP-43 proteinopathy that shows robust association with *TMEM106B* polymorphism. LATE patients present an amnestic dementia syndrome that resembles AD [19], and autopsy studies have revealed that LATE patients show prevalent TDP-43 proteinopathy, some with concurrent hippocampal sclerosis. All subtypes of LATE with distinct clinical patterns are associated with *TMEM106B* rs1990622 polymorphism (OR = 3.3), suggesting that *TMEM106B* serves as an independent risk factor for LATE [2].

*TMEM106B* polymorphism showed no or weak association with the risk for ALS [5], AD [20] or PD [4]. Interestingly, the leading brain proteinopathy in AD and PD patients are not TDP-43 (namely, amyloid plaques and neurofibrillary tangles in AD and α-synuclein in PD) [21]. Regarding ALS, although TDP-43 is the major protein deposition, the primary symptom of motor dysfunction is likely due to the damage of motor neurons in the spinal cord [22, 23]. These data strongly suggest that the pathological effect of *TMEM106B*’s polymorphism can be modulated by the interaction with TDP-43 in the brain. While the molecular mechanism underlying this interaction is currently unknown, one possibility is that TMEM106B facilitates the aggregation of TDP-43 in the brain [24], which causes downstream cytotoxicity [25–27].

If the scenario described above is true, TMEM106B could still contribute to the brain function decline in AD, PD and ALS, even though the primary clinical symptoms of these diseases are not related to the TDP-43 depositions in the brain. Consistent with this view, several SNPs of *TMEM106B* have been reported to correlate with cognitive decline in AD, PD and ALS. Specifically, *TMEM106B* protective alleles rs1990622C are associated with slower deterioration of language function in ALS patients [5]. In AD, analysis showed *TMEM106B* regulates genetic pathways that converge with those affected by APOE-amyloid-β interaction [28]. *TMEM106B* rs1990621 variation has also been reported to correlate with neuronal proportion [3]. *TMEM106B* rs1990621 variation has also been reported to correlate with levels of neurofilament light chain in the cerebrospinal fluid of AD patients [29], which is a strong indicator of neurodegeneration. Among PD patients, rs1990622T carriers exhibit faster longitudinal decline in cognition, indicating that *TMEM106B* functions as a genetic modulator for cognitive trajectory in PD [4].

Together, these studies of genetics showed that TMEM106B variants are associated with the onset or clinical manifestation of major neurodegenerative diseases, highlighting the importance of the interaction with TDP-43 in the brain. Next, we turn to examine the structural features of TMEM106B aggregates found in recent pathology studies.

#### TMEM106B protein depositions exist in a wide range of neurodegenerative diseases

**Fig. 1:**
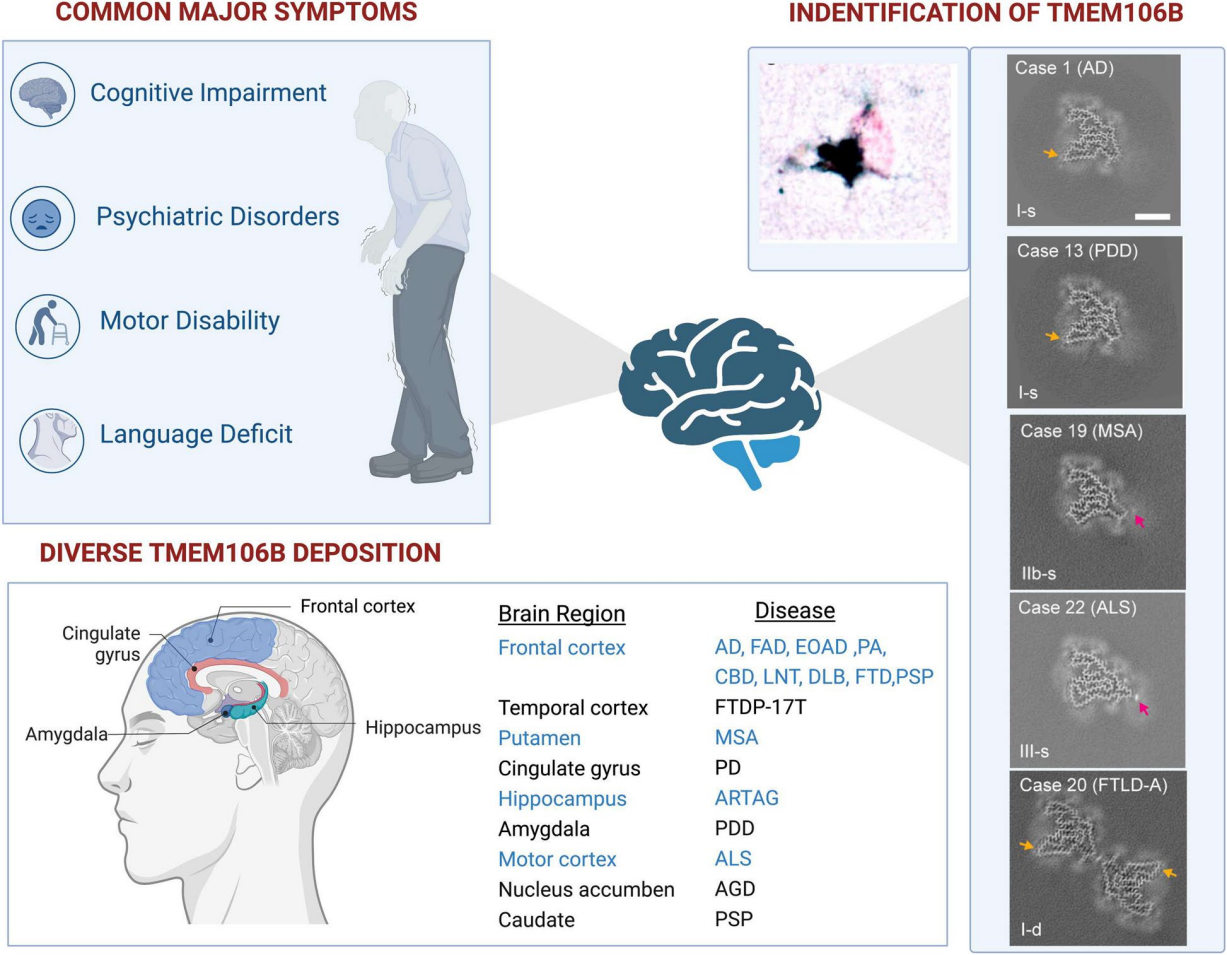
TMEM106B deposits in diverse brain regions and neurodegenerative diseases with different structural isoforms. Neurodegenerative diseases show overlapped clinical manifestation. TMEM106B has been reported as a common risk gene among those representative diseases, especially in those in which TDP-43 deposits are the main proteinopathy in the brain. Recent studies identified homotypic intracellular TMEM106B amyloid deposits in diverse brain regions from subjects with neurodegenerative diseases. Based on cryo-EM, TMEM106B filaments structure has three isoforms (type I, type II, type III). AD: sporadic Alzheimer’s disease; FAD: familial Alzheimer’s disease; EOAD: sporadic early-onset Alzheimer’s disease; PA: pathological aging; CBD: corticobasal degeneration; LNT: limbic-predominant neuronal inclusion body 4R tauopathy; DLB: dementia with Lewy bodies; FTD: frontotemporal dementia; PSP: progressive superanuclear palsy; FTDP-17 T: familial frontotemporal dementia and parkinsonism linked to chromosome 17 caused by MAPT mutations; MSA: multiple system atrophy; PD: sporadic Parkinson’s disease; ARTAG: aging-related tau astrogliopathy; PDD: Parkinson’s disease Dementia; ALS: amyotrophic lateral sclerosis; AGD: argyrophilic grain disease; FPD: familial Parkinson’s disease; Reproduced with the permission of Schweighauser, M. et al., [9]

Jiang et al. serendipitously discovered that all amyloid fibrils isolated from post-mortem brain tissues of four FTLD-TDP patients showed negative labeling of anti-TDP-43 antibody [10]. Subsequent immunogold labelling and cryo-electron microscopy revealed that these amyloid fibrils were made up with TMEM106B [10]. This finding was unexpected as TMEM106B was not previously reported to form amyloid fibrils. Several other studies also identified TMEM106B fibrils in diverse neurodegenerative diseases such as FTLD-TDP, tauopathy, AD, and α-synucleinopathy [7–9], and normal aging [9], indicating that TMEM106B fibrils are a previously unappreciated common protein aggregation that exists in the brain (Fig. 1). Interestingly, when compared to age-matched controls, the burden of TMEM106B fibrillization is much higher in most individuals with neurodegeneration [7, 9, 30], suggesting that the TMEM106B fibrils may not be a benign structure, but could exert a toxic effect and contribute to age-dependent neurodegeneration, although no direct evidence currently exists.

TMEM106B depositions have been identified in different brain regions. Frontal lobe area is the most common region to date to show TMEM106B deposits. Studies have reported TMEM106B filament in frontal lobe in patients with familial AD, early onset AD, PD, FTLD-TDP, corticobasal degeneration, limbic-predominant neuronal inclusion body 4R tauopathy, dementia with Lewy bodies, progressive superanuclear palsy and normal aging [7–10]. In addition, deposits have been reported in the motor cortex of ALS patients [9]. TMEM106B fibrils can also form in subcortical regions, including nucleus accumbens, hippocampus, cingulate gyrus, amygdala, putamen and caudate [7]. Overall, many aspects of the TMEM106B pathology remain unknown, probably due to limited post-mortem brain tissue from patients. At this point, while several studies reported that TMEM106B fibrils do exist in many brain regions, whether there is a region- or diseases- specific susceptibility for TMEME106B aggregation and how these aggregations evolve or spread longitudinally with the development of the diseases, require further investigation.

Cryo-EM studies have resolved that the TMEM106B fibrils are aggregates from the C-terminus fragments (S120-G254) [7–10]. Furthermore, researchers identified three major isoforms of TMEM106B fibrils. The three isoforms differ in their structure of the middle region (A167 – M210), in which type I forms a loose amphipathic cavity, and type II and type III form increasingly tighter structures. In addition, the same fibril isoform can connect to each other via the sidechains of residues K178 and R180 to form doublet fibril [7]. In most cases, the ratio between singlet and doublet fibrils varies between 0.5 and 2. Importantly, the type II and type III fibrils are particularly enriched in patients with neurodegenerative diseases, while type I and II can be found in brains with normal aging [8]. These structural data again indicated that a distinct isoform of TMEM106B may be associated with neurotoxicity [8].

### Physiological function of TMEM106B in regulating lysosome functions

#### Introduction of TMEM106B

TMEM106B is a single-pass, type 2 integral membrane glycoprotein with 274 residues that predominantly locates in the membranes of late endosomes and lysosomes [31]. TMEM106B shows robust colocalization with the late endosome and lysosome markers Rab7, cathepsin D, and LAMP1, and relatively poor colocalization with the early endosome marker Rab5 and the recycling endosome marker Rab11, indicating that late endosomes and lysosomes are the primary subcellular location of TMEM106B [6].

TMEM106B protein sequence contains three structural domains. The N-terminal region consists of residues 1–96, which extends into the cytoplasm, can interact with both itself and TMEM106C, forming homopolymer or hetero-multimers at the lysosome surface [32]. A single-pass helix transmembrane domain can be found at residues 97–117. A C-terminal region of residues 118–274 exists in the lumen of lysosomes. This domain contains five important N-glycosylation sites. For TMEM106B to be transported outside of the endoplasmic reticulum and into late-stage cellular compartments, glycosylation is necessary. Whether endogenous and transgenic overexpressed TMEM106B, they all localize to late endosomes and lysosomes [31]. Post-translational modifications have been reported on TMEM106B to modulate its function. Glycosylation of residues N183 and N256 have been reported to regulate lysosome localization and TMEM106B degradation [31]. In addition, the proteolytic processing of TMEM106B also occurs in the C-terminal region, which produces the fragment that forms the protein aggregation [30, 33].

#### TMEM106B regulates morphology, acidification and transport of lysosome

Previous studies reported that TMEM106B could regulate various aspects of lysosome functions. The N-terminal region of the protein can interact with the clathrin heavy chain (CTLC), the μ1 subunit of adipocyte protein 2 (AP2M1), and endocytic adaptor proteins, indicating that TMEM106B may be crucial for the endolysosome sorting process [32]. In addition, TMEM106B N-terminus can also interact with microtubule-associated protein 6 (MAP6), suggesting an important function in controlling the retrograde transport of lysosomes [34]. The C-terminal region of TMEM106B can interact with lysosomal protease cathepsin D [35] and proton pump V-ATPase [36], thus pointing to a modulatory role of TMEM106B for lysosome acidification and protein degradation.

**Fig. 2:**
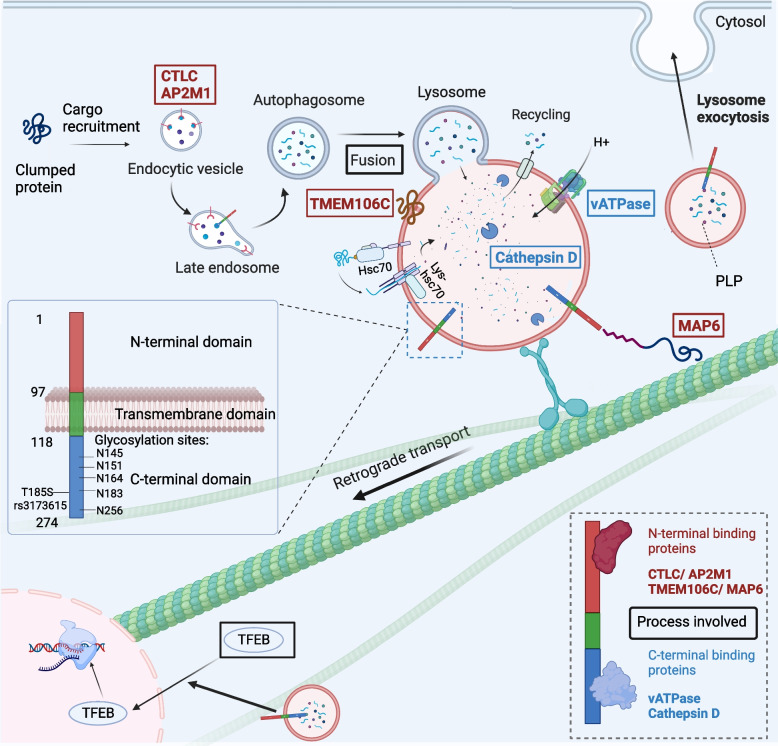
The physiological roles of TMEM106B under healthy conditions. TMEM106B is a type II transmembrane protein located on late endosome and lysosome, which has 274 residues and three structural domains. This C-terminal fragment (118–274 residue) contains five important N-glycosylation sites (N145, N151, N164, N183, N256). Proteins with known interaction with N-terminal TMEM106B are circled in red and C-terminus in blue. TMEM106B interacts with AP1 and v-ATPase V0 domain subunits, which controls the acidification of lysosomes. The interaction with MAP6 regulates lysosome retrograde transport. TMEM106B further activates TFEB-dependent lysosome biogenesis. Lysosomal activity is significantly modulated by intraluminal pH in addition to enzyme concentration and lysosome location within the cell to correctly degrade the contents. Endosome and lysosome fusion is also mediated by TMEM106B. TMEM106B plays important roles in the transportation, maturation and biogenesis of lysosomes. TMEM106B regulates lysosome traffic and exocytosis to affect PLP to be transported to the myelin sheath in oligodendrocytes

Consistent with these biochemistry characterizations, TMEM106B knockout led to a severe disruption of lysosome functions. TMEM106B knockdown reduces the total number of lysosomes in the cells [32]. The remaining lysosomes change from the normal cytoplasmic localization to an abnormal clustering at the axon initial segment or perinuclear space [32, 37]. At the same time, the morphology of lysosomes dramatically enlarges into a vacuole-like shape when lacking TMEM106B [38]. This is accompanied by a disruption of lysosome maturation, as TMEM106B knockdown resulted in less efficient fusion with autophagosome, poor protein degradation efficiency, and insufficient acidification [36, 39]. Thus, TMEM106B plays important roles in the transportation and maturation of lysosomes and is necessary for the physiological biogenesis of this organelle (Fig. 2).

#### Physiological processes regulated by TMEM106B

The *Tmem106b* knockout models show several pathological phenotypes, which may be ultimately attributed to altered lysosomal functions. In cultured neurons, lacking TMEM106B causes an imbalance between retrograde and anterograde transportation of lysosomes, leading to an abnormal accumulation of lysosomes around the soma. Blocking the overly active retrograde transportation can partially reverse the reduction of dendritic branching in cultured cells, indicating a restoration of lysosomal function. The general reduction of lysosome function in *TMEM106B* knockdown is also indicated by a build-up of lipofuscin [37], and an alteration of TFEB-related genetic pathways [40]. Importantly in mice, the hippocampus and cerebellum are the brain regions with high levels of Tmem106b expression in the brain [41], thus these two regions might be the most susceptible to the loss of TMEM106B function. Indeed, in Tmem106b knockout mice, the most severe disruption of lysosome function and cytotoxicity happens in the Purkinje cells, while cortical neurons show only mild changes [42]. In humans, the expression of *TMEM106B* is more universal cross different brain regions [43], suggesting that lacking TMEM106B may affect more broadly.

In addition to neurons, TMEM106B is also expressed in oligodendrocytes. TMEM106B deficiency results in myelination abnormalities including the separation and vacuolization of myelin sheaths [35]. At the same time, the numbers of matured oligodendrocytes are reduced [44]. The abnormal myelination in Tmem106b knockout mice may also be due to the change in lysosome functions. This is because the integration of major protein components, proteolipid protein (PLP) and myelin oligodendrocyte glycoprotein (MOG), to myelin sheath requires lysosome exocytosis [45, 46]. Lacking functional TMEM106B disrupts the normal trafficking of PLP to cell surface and induces perinuclear clustering of lysosomes [35], indicating a disruption of oligodendrocyte maturation [44]. *Tmem106b* knockout also leads to a general reduction in lysosomal proteins including cathepsin D in oligodendrocytes [35], which is known to be important for PLP processing [47].

While other glia cells also express Tmem106b, including astrocytes, microglia and endothelia cells, the function of Tmem106b in these cells are less clear. In Tmem106b knockout mice, no obvious lysosomal phenotypes were observed in microglia and astrocyte in the young Tmem106b^−/−^ mice [42], suggesting a different protein may exist in these cells to regulate lysosome biogenesis. Also, there is an upregulation of inflammatory genes in Tmem106b knockout mice, indicating that astrocytes and microglia could be activated [39]. In another study, TMEM106B deficiency in mice lead to reduced microglia proliferation and activation and increased microglial apoptosis in response to demyelination [48]. It is possible that the inflammatory phenotype in these cells is due to a secondary response to the ongoing cytotoxicity in neurons and oligodendrocytes.

### Disease-associated variants increase TMEM106B levels

#### *TMEM106B* variant increase TMEM106B level

The most well-studied TMEM106B variant rs1990622 is located in the non-coding region of the gene, which does not change the sequence or structure of the protein product. A recent study revealed that this region binds to a transcription factor CTCF (CCCTC-binding factor) and regulates the activity of TMEM106B promoter [49]. The disease-promoting variant rs1990620A increases the expression of TMEM106B [49]. Thus, it is possible that increasing levels of TMEM106B might be contributing to the higher risks for developing neurodegenerative diseases [1, 43]. This view is also supported from the studies examining the effect of another important SNP rs3173615, which affects the 185^th^ amino acid of the protein. This position is close to sites of glycosylation in the C-terminal region of the protein, which are important for the localization of TMEM106B to lysosomes [31]. Studies revealed that expressing the risk isoform T185 leads to a higher level of TMEM106B compared to that when expressing the protective isoform S185 [50]. Interestingly, suppressing lysosomal digestion blocks this effect, while stopping novel protein synthesis does not, indicating that the risk isoform T185 reduces the degradation of the protein, leading to a higher level of TMEM106B in cells [50]. Together, these studies link the elevated levels of TMEM106B with higher risk for developing neurodegenerative diseases.

#### Increasing *TMEM106B* levels disrupt lysosome function

The findings that higher levels of TMEM106B are associated with higher risk for developing neurodegenerative diseases suggests that the overexpression of TMEM106B might be a plausible model to study its involvement in the pathogenesis process. Interestingly, TMEM106B overexpression disrupts several aspects of lysosome function, similar to the phenotypes found with TMEM106B deficiency. In cell cultures, over expression of TMEM106B leads to the formation of abnormally large vacuoles and associated reduction in function [35, 51, 52], which are associated with reduced dynamism [32], less effective protein degradation [51] and poor acidification of lysosomes [35, 51]. Furthermore, humans carrying the rs1990622 risk allele (T/T) showed increased Purkinje neuron loss, mimicking the phenotype seen in TMEM106B knockout mice [42]. Bcl-xL, which is previously used for preventing caspase-dependent mitochondrial-mediated apoptosis, was proved to significantly ameliorate the neurotoxicity of TMEM106B overexpression [53]. Together, these data indicate a similar effect of lysosome dysfunction between TMEM106b deficiency and overexpression.

#### Increased TMEM106B levels could potentially promote its aggregation

Why would TMEM106B deficiency and overexpression both disrupt lysosome function? A previous hypothesis suggests that TMEM106B level is critical for normal lysosome function and an imbalance towards either direction could disrupt this delicate equilibrium. In a *Tmem106b* transgenic mouse model, total protein levels of TMEM106B remained unchanged despite increased amount of mRNA and protein expression [54]. While this provides evidence supporting tight control of TMEM106B in vivo, the molecular mechanism allowing deficiency and overexpression to show the same phenotype is still lacking.

**Fig. 3:**
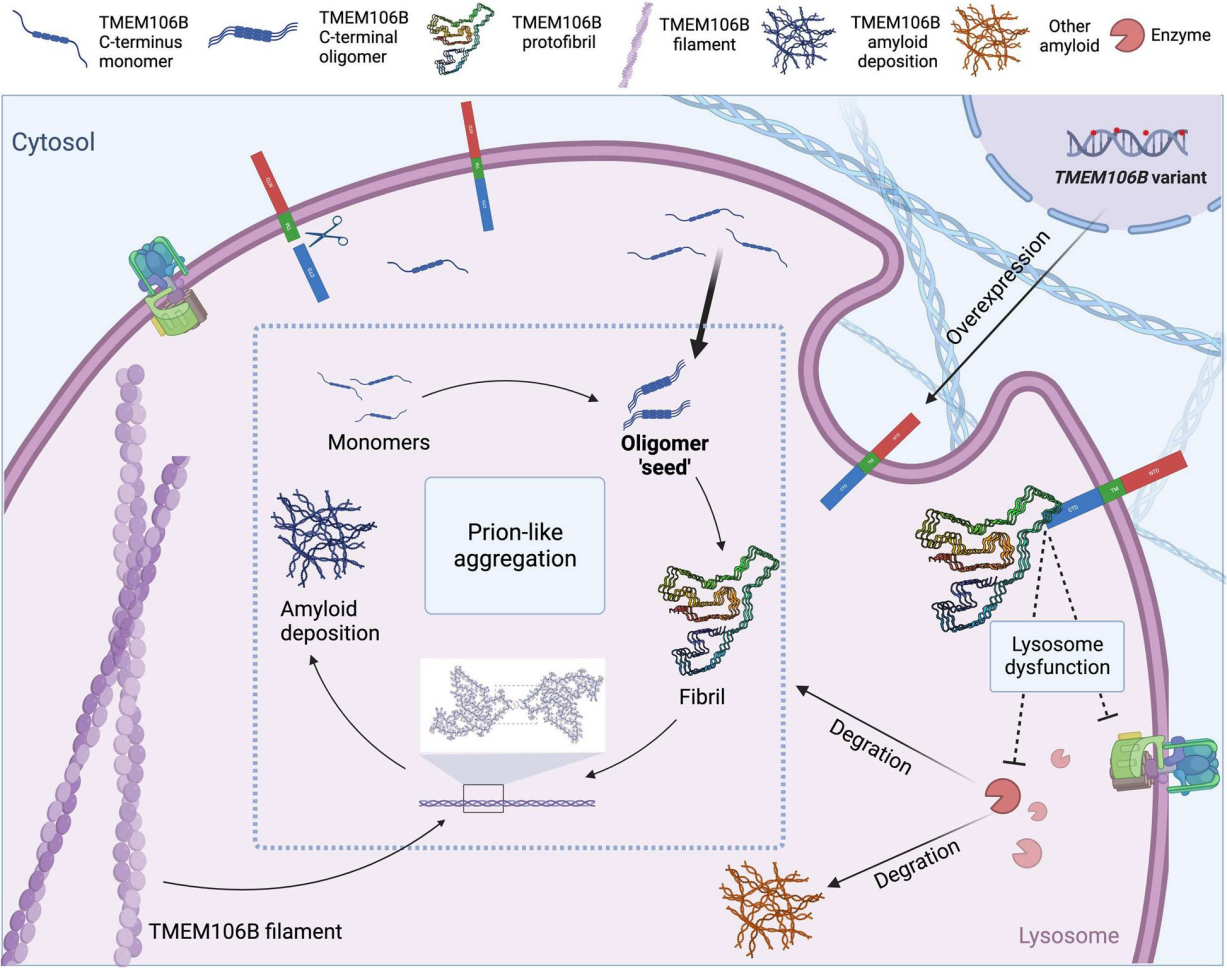
Disease-associated variants increase TMEM106B levels and induce prion-like aggregation. Genetic variants of *TMEM106B* cause overexpression of TMEM106B protein. TMEM106B 118–274 residue is cleaved by a protease, and the resulting C-terminal fragment is prone to aggregate. When lysosome function is insufficient, or the production of TMEM106B increased, C-terminal fragments reach the critical point of fibrilization. Cryo-EM micrographs showed that TMEM106B filaments are comprised of two protofilaments. The TMEM106B oligomers serving as ‘seeds’ will recruit or interact with normal TMEM106B proteins, interfering their functions in modulating lysosome biogenesis, thus effectively causing a deficiency of functional TMEM106B. The prion-like aggregation develops into detectable TMEM106B deposits eventually. The aggregation of TMEM106B(120–254) into fibrils further hinders the normal function of TMEM106B protein on the membrane, leading to lysosomal dysfunction, which promotes the accumulation of other pathological protein aggregates such as those formed by TDP-43, tau or a-synuclein and the free TMEM106B fragments(monomer, oligomer, fibril) in the lysosome. Reproduced with the permission of Schweighauser, M. et al., [9]

In light of the recent findings that TMEM106B can form protein aggregates commonly seen in patients with neurodegenerative diseases, we here propose a model that reconciles the phenotype of TMEM106B deficiency and overexpression. The proteolytic processing of TMEM106B produces a C-terminal fragment in the lumen of lysosomes. In normal conditions, this fragment undergoes a lysosome-dependent degradation. However, when lysosome function is insufficient, or the levels of TMEM106B increased, the C-terminal fragments of TMEM106B will not be degraded in time, and can start the process of fibrilization. The TMEM106B fibrils may recruit or interact with normal TMEM106B proteins, interfering their functions in modulating lysosome biogenesis, thus effectively causing a deficiency of functional TMEM106B (Fig. 3).

Consistent with this hypothesis, previous studies have shown that lysosome activity can regulate the levels of TMEM106B. For example, acute treatment of Bafilomycin reduced lysosomal activities by interfering with lysosome acidification [55, 56]. Applying Bafilomycin significantly increased TMEM106B on protein levels through a predominant post-transcriptional mechanism [31]. Increased levels of TMEM106B are also reported in models of lysosomal storage disorder [57].

While our model remains speculative at present, several testable hypotheses can be made. A key process that links TMEM106B overexpression with a loss-of-function phenotype is a fibrilization process. We hypothesize that electron microscopy examination of tissue or cells with elevated expression of TMEM106B could reveal the existence of TMEM106B fibrils. In addition, we propose that TMEM106B fibrils could have ‘prion-like’ properties that recruits monomers. This is testable in vitro by seeding the TMEM106B monomer solution with extracted fibrils and examining the fibrilization over time. Furthermore, we propose that introducing TMEM106B fibrils will disrupt lysosome functions, which is readily testable in cultured cells. These experiments could provide mechanistic insights linking the overexpression phenotype due to disease-associated TMEM106B mutations and the TMEM106B fibrils universally found in patients’ brains.

### Other risk factors interact with TMEM106B pathology

#### The association with aging

*TMEM106B* gene expression is associated with normal aging [58]. Recent studies demonstrate that TMEM106B forms amyloid fibrils in human brains in an age-dependent manner [7–10]. In addition, in patients with neurodegenerative diseases, the amount of TMEM106B fibrils are found to be higher than age-matched control brains [7]. These data indicate that the formation of TMEM106B fibrils exacerbated with aging, and can be additionally modulated by disease conditions. However, it is not clear whether the increased amount of TMEM106B fibrils in diseased brains is an indication of reduced lysosomal functions, or a cause for lysosomal dysfunction. It has been reported that aging is associated with reduced lysosomal acidification and protease activity [59, 60], which could contribute to the insufficient degradation of TMEM106B C-terminal fragment. On the other hand, the formation of TMEM106B fibrils, according to our model (Fig. 3), may be in turn depleting the endogenous TMEM106B and exacerbates defects in lysosome biogenesis. This vicious cycle could contribute to the accumulation of other protein aggregates in the cell, eventually leading to complete failure of the lysosome function and cell death.

#### The association with Progranulin (*GRN* mutation)

One of the major neurodegenerative diseases associated with TMEM106B is frontotemporal dementia (FTD). The loss-of-function mutation of the *GRN* is well-characterized to cause familial FTD [61]. Importantly, the risk for FTD in individuals with *GRN* mutations is further modulated by SNPs in the *TMEM106B* gene [15, 17], suggesting an additive effect of the mutations in these two genes. Since localization to lysosomes is important for progranulin function [62] and loss of progranulin could lead to lysosome storage disease [63, 64], it is possible that *TMEM106B* and *GRN* mutations both contribute to the development of FTD via worsening lysosome functions. Interestingly, a study showed that Tmem106B deletion and *Grn* deletion cause opposite changes in lysosome proteins, thus knocking out Tmem106b to a relatively low level could partially rescue the phenotype of *GRN* knockout mice [36]. However, this result remains controversial as several studies showed that deletion of *Tmem106b* on a background of *GRN* knockout further disrupts lysosome/autophage function as well as the health of the animal [39, 65, 66]. Furthermore, another study reported no benefitial effect of partial Tmem106b reduction on the social deficits and most lysosome abnormalities in Grn^+/−^ mice [67]. Also knocking in the classic protective Tmem106b^T186S^ variant (SNP rs3173615) did not exert protective effects in *GRN* knockout mice [68]. The inconsistent results regarding whether modulating TMEM106B levels is a viable therapeutic strategy for GRN-FTLD call for more studies.

### Future direction based on TMEM106B pathology and therapy

#### TMEM106B is involved in development of neurodegenerative disease

Prior genetics studies revealed a robust relationship between several SNPs in the *TMEM106B* gene and the risk for developing neurodegenerative diseases. However, the biological mechanism underlying this association remains mysterious, since studies showed that TMEM106B is necessary for lysosome biogenesis, yet the disease-associated genotypes all lead to increased levels of TMEM106B. Given the recent reports that TMEM106B can form fibrils in the brain [7–10], we proposed a new model in this review as a plausible pathogenic mechanism: increased levels of TMEM106B may promote the formation of TMEM106B fibrils, which exerts a dominant negative effect on the endogenous TMEM106B and disrupts lysosome function. As described in the previous section, several specific predictions derived from this model can be tested and may elucidate the pathogenic mechanism of TMEM106B mutations.

**Fig. 4:**
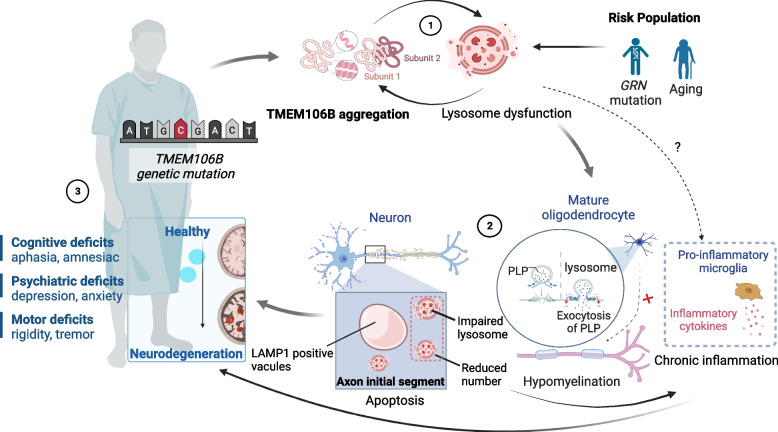
Overall description of TMEM106B related disease pathology. TMEM106B and lysosome dysfunction forms a vicious cycle. TMEM106B deficiency triggers abnormal lysosome manifestations in neuron: reduced number, abnormal localization at the axon initial segment or perinuclear space and a vacuole-like shape. TMEM106B abnormal aggregation also induces hypomyelination by oligodendrocyte, further activating microglia-related neuroinflammation, disrupting signal transduction and related physiological process and driving the cognitive, psychiatric, and motor lesions in diverse neurodegenerative diseases. Other risk factors like aging or *GRN* mutation also contribute to this cycle via affecting lysosome functions

Furthermore, under the framework of our model, lysosome dysfunction due to TMEM106B mutations could add to the formation of other protein aggregation, such as amyloid-β, α-synuclein, phosphorylated tau and TDP-43. Interestingly, genetics studies showed that TMEM106B mutations is particularly detrimental for diseases with TDP-43 being the primary type of protein aggregates in the brain (see Sect. "Mutations in TMEM106B are risk factors for diverse neurodegenerative diseases"), suggesting that the effect of TMEM106B mutation may have certain specificity that preferentially affects TDP-43 over other types of protein aggregation. Consistent with this, TMEM106B genotype has been proven to modify TDP-43 pathology independent of C9orf72 status in human cohorts and cellular model [24]. The mechanism underlying such specificity requires future investigations (Fig. 4).

#### Therapeutic opportunities

**Fig. 5:**
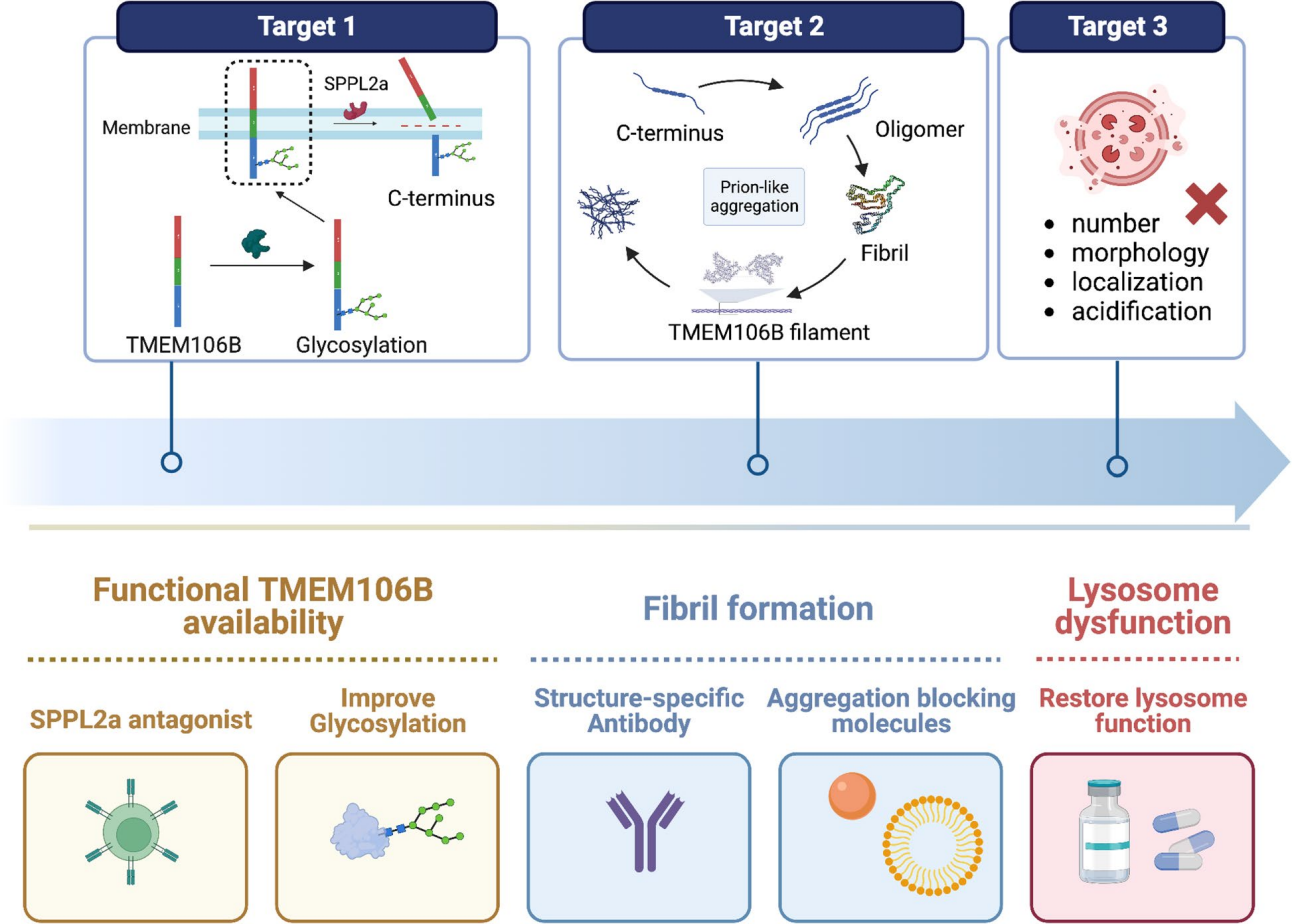
Multiple therapeutic strategies targeting TMEM106B for intervention. We proposed that the formation of TMEM106B fibrils may be the key turning point that initiates the downstream pathogenic cascade. Several TMEM106B- or lysosome-based therapeutic approaches have been proposed, including inhibitors of cleavage, improvement of TMEM106B glycosylation, immunotherapy using antibodies against oligomer fibrils, C-terminus aggregation inhibitors and stabilization of lysosome

To date there are no studies that have targeted TMEM106B for therapeutic intervention. This is understandable as the pathogenic mechanism of TMEM106B is not completely known. It is questionable whether increasing or decreasing the levels of TMEM106B could offer therapeutic effects. Our hypothesized model could provide some new insights in this regard. We proposed that the formation of TMEM106B fibrils might be the key turning point that starts the downstream pathogenic cascade. Therefore, targeting this initial fibrilization process could be a viable therapeutic target. Specifically, we suggest the following strategies (Fig. 5).

#### Improve glycosylation

For TMEM106B locating accurately to late-stage cellular compartments, glycosylation is necessary. Post-translational modifications could mediate the structures of polymorphic fibrils by influencing their inter-protofilament interfaces [69], although currently direct biochemical measurements of fibrilization kinetics affected by glycosylation is lacking for TMEM106B. On the other hand, glycosylation guarantees the protein stability and degradation rate [70], which could change the availability of physiologically functional TMEM106B. Therefore, improving post translational glycosylation is a potential therapeutic approach.

#### Blocking the production of C-terminal fragment

The C-terminal fragment of TMEM106B is the monomer to form fibrils. Thus, suppressing the production of this fragment could be an effective way of blocking the fibrilization process. To preserve the homeostasis of membrane proteins, single pass transmembrane proteins undergo sequential processing that includes ectodomain shedding and intramembrane proteolysis. This fragment of TMEM106B is produced by lysosome proteases in the luminal domain followed by an intracellular cytosolic domain is produced by the signal peptide peptidase-like 2A (SPPL2a) family protease in the transmembrane area [71]. SPPL2a antagonist could be a target to specifically block the production of C-terminal fragment. Despite the fact that proteasomes typically work better for non-aggregated proteins, inclusions can still be eliminated if abnormal protein fragment creation is halted [72].

#### Interfering with the fibrilization process

It is possible that certain small molecules could block the β-sheet formation of the TMEM106B fibrils. This approach could prevent the disruption of the endogenous TMEM106B’s function, thus blocking the disruption of lysosome biogenesis.

#### Promoting the degradation of TMEM106B fibrils

Conformation-specific antibodies for the TMEM106B fibrils can be raised to facilitate the degradation of TMEM106B fibrils. However, since the antibody-mediated degradation likely depends on microglia in the brain, this approach maybe more effective for the clearance of extracellular TMEM106B fibrils. Therefore, this strategy might be preventing the spreading of the TMEM106B aggregation between cells, but have little effects in neurons already containing these fibrils.

#### Restoring lysosome function

Given that normal TMEM106B maintain the physiological processes of lysosome, TMEM106B pathology is often associated with a breakdown of lysosome stability. A recent study found that lysosome function could be repaired, and this process is heavily reliant on lysosomal membrane integrity via complex regulation [73]. Repairing the proper pH circumstance and proteolysis function serves as a promising strategy for halting the progress of disease.

## Conclusions

In this review, we propose a novel mechanism for neurodegenerative diseases inspired by the widely identification of TMEM106B deposition. Genetic mutations trigger the aggregation of TMEM106B filaments and imbalance of lysosome physiology, aggravated by advancing age, genetic predisposition, and environmental factors. Lysosome dysfunction further aggravates TMEM106B accumulations. The collapse of cellular autophagy mechanisms result in not only hallmark protein aggregation but also blocked signaling and even apoptosis independently. This cycle is widely distributed in neurons and glial cells among all brain regions. Targeting these amyloid fibrils could be a promising strategy for restoring neuron or glia functions, delaying the progress of neurodegeneration. Since the structures of TMEM106B filament vary between subtypes of diseases, the conformational variations could be an indicator for disease progress. This model for choosing which patients will benefit most from early therapies targeting the lysosome in a precision medicine approach needs more precise evidence before it can be established.

## Data Availability

Not applicable.

## Abbreviations

AD: Alzheimer’s disease
FAD: Familial Alzheimer’s disease
FTLD: Frontotemporal lobar degeneration
ALS: Amyotrophic lateral sclerosis
MSA: Multiple system atrophy
SNP: Single-nucleotide polymorphism
TDP-43: TAR DNA-binding protein 43
CTLC: Clathrin heavy chain
AP2M1: The μ1 subunit of adipocyte protein 2
MAP6: Microtubule-associated protein 6
PLP: Proteolipid protein
MOG: Myelin oligodendrocyte glycoprotein
LATE: Limbic-predominant age-related TDP-43 encephalopathy
EOAD: Sporadic early-onset Alzheimer’s disease
PA: Pathological aging
CBD: Corticobasal degeneration
LNT: Limbic-predominant neuronal inclusion body 4R tauopathy
DLB: Dementia with Lewy bodies
FTD: Frontotemporal dementia
PSP: Progressive superanuclear palsy
FTDP-17 T: Familial frontotemporal dementia and parkinsonism linked to chromosome 17 caused by MAPT mutations
MSA: Multiple system atrophy
PD: Parkinson’s disease
ARTAG: Aging-related tau astrogliopathy
PDD: Parkinson’s disease Dementia
AGD: Argyrophilic grain disease
FPD: Familial Parkinson’s disease
SPPL2a: Signal peptide peptidase-like 2A
